# The burden of headache disorders in Nepal: estimates from a population-based survey

**DOI:** 10.1186/s10194-016-0594-0

**Published:** 2016-01-25

**Authors:** Kedar Manandhar, Ajay Risal, Mattias Linde, Timothy J. Steiner

**Affiliations:** Department of Neuroscience, Norwegian University of Science and Technology, Edvard Griegs Gate, Trondheim, NO 7489 Norway; Dhulikhel Hospital, Kathmandu University Hospital, Kavre, Dhulikhel, Nepal; Norwegian Advisory Unit on Headache, St Olavs University Hospital, Trondheim, Norway; Division of Brain Sciences, Imperial College London, London, UK

**Keywords:** Migraine, Tension-type headache, Medication-overuse headache, Public health, Population-based study, Burden of disease, Disability, Nepal, South-East Asia region, Global campaign against headache

## Abstract

**Background:**

Headache disorders, particularly migraine and tension-type headache (TTH), are among the most prevalent global public-health problems. Medication-overuse headache (MOH) is a common sequela of mismanagement of these. Migraine and MOH are highly disabling. Formulation of responsive health policy requires reliable, locally-derived, population-based data describing both individual and societal impact of headache disorders. South-East Asia is the only one of WHO’s six world regions in which no such national data have yet been gathered.

**Methods:**

In a nationwide population-based cross-sectional study, a representative sample of Nepalese-speaking adults (18–65 years) were randomly selected by stratified multistage cluster sampling. Trained interviewers made unannounced door-to-door visits and enquired into headache and its attributable burden using a culturally-adapted and validated Nepalese translation of the Headache-Attributed Restriction, Disability, Social Handicap and Impaired Participation (HARDSHIP) questionnaire.

**Results:**

Among 2100 participants, 1794 (85.4 %) reported headache during the preceding year (male: 689 [38.4 %], female 1105 [61.6 %]; mean age 36.1 ± 12.6 years). Mean headache frequency was 3.8 ± 6.2 days/month, mean headache intensity 2.1 ± 0.7 on a 0–3 scale, and mean attack duration 41.9 ± 108.5 h. All aspects of symptom burden (frequency, intensity and duration) were greater among females (*p* < 0.001). Participants with headache had poorer quality of life (QoL) than those without (*p* < 0.001); QoL was worst among those with probable MOH (pMOH).

Mean proportions of total available time spent in the ictal state were 5.4 % among participants with migraine, 3.9 % among those with TTH and 44.7 % among those with pMOH, with headache-related disabilities of 2.4, 0.15 and 9.7 % respectively. At population level, these disorders were responsible for reduced functional capacities of 0.81, 0.06 and 0.20 %. Total lost productive time due to headache was 6.8 % for the 85 % of the population with headache. Males lost more paid worktime than females (*p* < 0.001); the reverse was so for household worktime (*p* < 0.001).

**Conclusions:**

Headache disorders, very common in Nepal, are also highly burdensome at both individual and population levels. There is a substantial penalty in lost production. The remedy lies in better health care for headache; structured headache-care services are urgently needed in the country, and likely to be cost-saving.

## Background

Primary headache disorders – migraine and tension-type headache (TTH) – are among the most prevalent diseases in the world [1–3]. These disorders, together with their major sequela, medication-overuse headache (MOH), are of substantial importance to public health nationally and globally because they lead to widespread ill health and impaired quality of life (QoL) and are disabling [4] The Global Burden of Disease Study 2013 (GBD2013) found migraine to be the sixth highest cause of disability worldwide, and MOH the 18th, measured in years of life lost to disability (YLDs) [3]. Collectively, headache disorders are the third highest cause of disability in the world [5]. The economic consequences through productivity losses are substantial [6].

Mitigating action is required, a message strongly endorsed by the World Health Organization (WHO) [4, 7]. But decisions about the allocation of national health-care resources are best informed by reliable, locally-derived, population-based data. This is especially important in developing countries such as Nepal, which have very constrained health budgets and a clear imperative to maximise health gain from them. In the case of headache care, this means data on the burden attributable to headache disorders harvested from the population of Nepal as the basis of needs assessment. Recent population-based studies in other developing countries [8–10] have shown high headache prevalences and heavy headache-attributable burden, but these factors remain poorly described in many large and populous areas of the world [2, 4] – nowhere more obviously than in the South-East Asia Region (SEAR). In fact, SEAR, in which Nepal lies, is the only one of WHO’s six world regions for which no national data of this type have yet been gathered in any country [4].

Nepal is one of the poorest countries within SEAR [11], but with great geographic and cultural diversity. Its location in the Himalayas, and their foothills and plains beyond, includes eight of the world’s ten highest peaks, among them Mount Everest, the highest point on Earth [11]. Its population is approximately 30 million [12], with a rather unequal distribution of wealth so that about one quarter live below the international poverty line [11]. The majority of Nepalese engage in agriculture [13]. More than 70 ethnic groups maintain different cultures and spoken languages [13].

We earlier reported headache prevalence data from our adult population-based study in Nepal [14]. The 1-year prevalence of migraine in this country (34.1 %) was uniquely high, while the point prevalence of probable MOH (pMOH) (2.1 %) was towards the upper end of the range for most countries studied [15]. The 1-year prevalence of TTH (44.1 %) was in line with the global average [2, 3]. Here, with the specific purpose of informing health policy, and as a study conducted within the Global Campaign against Headache, we report the estimates of burden attributable to these disorders in Nepal.

## Methods

### Ethics

The Nepal Health Research Council, the Institutional Review Committee of Kathmandu University School of Medical Sciences, Dhulikhel Hospital, and the Regional Committee for Health and Research Ethics in Central Norway all approved the study protocol.

All participants were informed about the nature and purpose of the study and this was documented in accordance with requirements of the three committees.

### Study design and sampling

The study design, sampling and data collection procedures have been reported in detail previously [16]. In summary, this was a cross-sectional survey using structured interviews administered by trained health workers making unannounced door-to-door visits during May, 2013. To obtain a nationally representative sample, we used multistage stratified cluster sampling in all three physiographic divisions of the country and, within each division, all five development regions (Far-Western, Midwestern, Western, Central and Eastern). We randomly selected one eligible adult (aged 18–65 years, Nepalese-speaking and living in Nepal) from each household.

### Instruments

We used the Headache-Attributed Restriction, Disability, Social Handicap and Impaired Participation (HARDSHIP) questionnaire developed by *Lifting The Burden* (LTB) for population-based studies [17]. The original English-language version was translated into Nepalese according to LTB’s translation protocol for hybrid documents [18] and modified according to Nepalese culture [19]. The questionnaire consisted of four parts relevant to this report. For all participants there were (i) personal and demographic enquiry and (ii) a neutral headache screening question (“Have you had a headache during the last 12 months?”). Those who answered “no” to the latter were classified as headache-free. Those who answered “yes” were asked whether their headaches were of one or more types and, if more than one, to focus only on the most bothersome type. Only those who answered positively to the screening question were also asked (iii) diagnostic questions in line with the International Classification of Headache Disorders (ICHD-3 beta) [20], and (iv) questions ascertaining various aspects of headache burden.

We measured symptom burden in terms of frequency (days/month), intensity (with response options “not bad”, “quite bad” and “very bad”, which we interpreted as mild, moderate and severe) and duration (hours).

We enquired into willingness to pay (WTP) as an overall measure of burden [17] using a bidding game method [21]. We asked how much participants would be willing to pay per month for an effective treatment such that their headaches would no longer bother them. Bidding began at NPR 100/month (at the time of the survey, NPR 100 ≈ USD 1): was the respondent willing to pay this amount? If “yes”, the interviewer incremented the bidding stepwise (NPR 200, 400, 1000 and 2000) until the answer was “no”, or it was clear that the participant would pay > NPR 2000. If the opening bid of NPR 100 was declined, the interviewer instead reduced stepwise (NPR 40 and 20) until the participant said “yes”, or it became clear that nothing would be paid. Finally, in every case, an exact amount was agreed upon as the individual’s WTP-value.

We assessed QoL using the World Health Organization Quality of Life-8 (WHOQOL-8) questionnaire [22]. Its eight items addressed subjective wellbeing and satisfaction in four domains (two in each): psychological, physical, social and environmental [17]. Each item was graded on a scale of 1–5, with a higher score indicating better QoL.

We estimated lost time due to headache during the preceding 3 months using the Headache-Attributed Lost Time (HALT) questionnaire [23]. Its first two questions asked for the numbers of days in that period (i) completely missed from paid work and (ii) with <50 % productivity because of headache while at work; the next two asked for numbers of days of household work (iii) completely missed and (iv) with <50 % productivity; the last enquired into (v) the number of days on which family, social or leisure activities were missed because of headache.

### Headache diagnosis

The diagnostic method has been described previously [14]. Diagnoses were not made during the interviews but later according to an algorithm. Participants reporting headache on ≥15 days/month were first separated as a distinct group because they cannot be fully diagnosed by questionnaire. Those who in addition were overusing acute medication were considered to have pMOH [24]; the remainder were categorised as “other headache on ≥15 days/month”. To all others, reporting headache on ≤14 days/month, the algorithm applied modified ICHD-3 beta criteria [20] in the following order: definite migraine, definite TTH, probable migraine and probable TTH. Definite and probable migraine were subsequently considered together, and likewise definite and probable TTH, for attribution of burden. The few remaining cases were unclassifiable.

### Statistics

Categorical variables are presented as numbers and percentages. Continuous variables are presented as means with standard deviations (SDs) and/or medians with interquartile ranges (IQRs).

We transformed the categorical variable for headache intensity (mild, moderate, and severe) into a numerical scale 0–3 (0 being no pain), and treated these results as continuous data.

We summed responses to the first four items (i-iv) of HALT to estimate total productivity loss. These scores were treated as continuous variables. We separately summed the first and second items (i, ii) to estimate lost paid worktime, and the third and fourth (iii, iv) to estimate lost household worktime.

We estimated individual and population-level disability attributable to migraine, TTH and pMOH using the disability weights (DWs) from GBD2013 [3] for the ictal states of each. We calculated the mean absolute time spent in the ictal state (T_abs_) as the product of mean attack frequency (AF) and mean attack duration (D). For migraine, because headache frequency (HF) had been recorded as days/month, we assumed participants with attacks lasting >24 h had counted days affected rather than number of attacks per month. Therefore, to calculate mean AF we made separate computations for those with attack durations of ≥24 h and <24 h, using the formula AF = HF/D in the former group. We then took the weighted mean of the two groups. We did not need to perform the same manipulation for TTH, because the mean duration of headache was <24 h (Table 1). For pMOH, we made separate calculations for those describing durations of <24 h and those asserting pain “never goes away”, for whom we assumed AF = 30 and D = 24; there were no respondents in between. Again we took the weighted mean. From T_abs_ (in hours/year) we calculated the proportion of total available time that was spent in the ictal state (T_pro_) by dividing by (24*365). We multiplied this proportion by the DW for the disorder in question to calculate individual disability (DIS_per_), and then by the prevalence of the disorder to arrive at population-level disability (DIS_pop_).

**Table 1:** Symptom burden: frequency, intensity and duration of headache for all headache and each headache type, by gender

	All	Male	Female	*p* ^a^
All headache (*N* = 1794 [males 689; females 1105])				
Headache frequency (days/month)				
Mean ± SD	3.8 ± 6.2	2.9 ± 4.8	4.3 ± 6.9	<0.001
Median [IQR]	2.0 [0.4–3.0]	1.0 [0.3–3.0]	2.0 [0.5–4.0]	<0.001
Headache intensity				
Not bad	373 (20.8)	184 (26.7)	189 (17.1)	
Quite bad	901 (50.2)	344 (49.9)	557 (50.4)	
Very bad	520 (29.0)	161 (23.4)	359 (32.5)	
Mean^b^ ± SD	2.1 ± 0.7	2.0 ± 0.7	2.2 ± 0.7	<0.001
Median^b^ [IQR]	2 [2, 3]	2 [1, 2]	2 [2, 3]	<0.001
Headache duration (hours)				
Mean ± SD	41.9 ± 108.5	23.3 ± 46.8	53.4 ± 131.9	<0.001
Median [IQR]	8.0 [2.0–45.0]	6.0 [1.3–24.0]	10.0 [2.3–48.0]	<0.001
Migraine (*N* = 728 [males 249; females 479])				
Headache frequency (days/month)				
Mean ± SD	2.3 ± 0.6	2.2 ± 2.3	2.4 ± 2.3	0.46
Median [IQR]	2.0 [0.4–3.0]	2.0 [0.3–3.0]	2.4 [0.6–3.0]	0.29
Headache intensity				
Not bad	60 (8.2)	21 (8.4)	39 (8.1)	
Quite bad	354 (48.6)	123 (49.4)	231 (48.2)	
Very bad	314 (43.1)	105 (42.2)	209 (43.6)	
Mean^b^ ± SD	2.3 ± 0.6	2.2 ± 0.6	2.4 ± 0.6	0.72
Median^b^ [IQR]	2 [2, 3]	2 [2, 3]	2 [2, 3]	0.71
Headache duration				
Mean ± SD	33.6 ± 47.9	29.7 ± 43.3	35.7 ± 50.0	0.093
Median [IQR]	12.0 [4.0–48.0]	10.0 [4.0–48.0]	14.0 [4.0–48.0]	0.084
Tension-type headache (*N* = 863 [males 384; females 479])				
Headache frequency (days/month)				
Mean ± SD	1.8 ± 0.6	1.6 ± 1.9	2.0 ± 2.3	0.009
Median [IQR]	1.0 [0.3–2.0]	1.0 [0.2–2.0]	1.0 [0.3–3.0]	0.011
Headache intensity				
Not bad	292 (33.8)	154 (40.1)	138 (28.8)	
Quite bad	477 (53.3)	198 (51.6)	279 (58.2)	
Very bad	94 (10.9)	32 (8.3)	62 (12.2)	
Mean^b^ ± SD	1.8 ± 0.6	1.7 ± 0.6	1.9 ± 0.6	<0.001
Median^b^ [IQR]	2 [1–3]	2 [1–2]	2 [1–2]	<0.001
Headache duration				
Mean ± SD	16.0 ± 31.0	14.0 ± 27.1	18.0 ± 33.6	0.043
Median [IQR]	4.0 [0.8–15.0]	4.0 [0.8–20.0]	4.0 [0.8–20.0]	0.030
Probable medication-overuse headache (*N* = 46 [males 11; females 35])				
Headache frequency (days/month)				
Mean ± SD	23.1 ± 6.2	19.4 ± 4.4	24.0 ± 6.3	0.010
Median [IQR]	20.0 [16.7–30.0]	17.0 [16.0–20.0]	30.0 [19.0–30.0]	0.063
Headache intensity				
Not bad	1 (2.2)	0 (0.0)	1 (2.9)	
Quite bad	15 (32.6)	6 (54.5)	9 (25.7)	
Very bad	30 (65.2)	5 (45.5)	25 (71.4)	
Mean^b^ ± SD	2.6 ± 0.5	2.5 ± 0.5	2.7 ± 0.5	0.65
Median^b^ [IQR]	3 [2–3]	2 [2–3]	3 [2–3]	0.23
Headache duration				
Mean ± SD	318.9 ± 309.6	91.0 ± 82.8	390.5 ± 320.6	0.21
Median [IQR]	176.0 [40.0–720.0]	48.0 [32.0–200.0]	375.0 [45.0–720.0]	0.020

Values (if not otherwise stated) are n (%); *SD* standard deviation, *IQR* interquartile range; ^a^comparing genders using Student’s *t*-test for difference between means and Mann–Whitney *U*-test for medians; ^b^mean and median on a scale of 0–3

We used Student’s *t*-test for significance of differences between means of two groups and one-way analysis of variance (ANOVA) for more than two groups. Since most of the continuous variables were skewed in distribution, we also used the non-parametric Mann–Whitney *U*-test or Kruskal-Wallis test to examine differences between groups. We considered *p* < 0.05 as statistically significant. All data were analysed with SPSS 21.0 software (SPSS Inc., Chicago, IL, USA).

## Results

We interviewed 2100 participants aged 18–65 years (participation rate >99 % [16]), of whom 1794 (85.4 %) reported headache during the preceding year (male: 689 [38.4 %], female 1105 [61.6 %]; mean age 36.1 ± 12.6 years; 686 [38.2 %] rural; 949 [52.9 %] living at altitude ≥1000 m). These 1794 were included in the analysis of burden; 728 [40.5 %] reported migraine, 863 [48.1 %] TTH, 46 [2.6 %] pMOH and 115 [6.4 %] other headache on ≥15 days/month.

### Symptom burden

Symptom burden is presented in Table 1 by headache type and gender. From a public-health perspective it was substantial: the mean overall reported headache frequency was 3.8 days/month – 1 day in every eight – and mean overall reported intensity was 2.1. Half of all participants with headache (901; 50.2 %) reported moderate headache and another nearly one third (520; 29.0 %) reported severe headache. By headache type, pMOH was of course the most frequent headache; it was followed by migraine, then TTH. The same rank order was seen for both headache intensity and duration. On every measure, females were worse affected than males with regard to all headache and TTH, but not significantly so with regard to migraine or (taking both means and medians into account) pMOH.

### Quality of life and willingness to pay

Participants without headache had significantly higher (*ie*, better) WHOQOL-8 scores than those with headache (median 29.0 [IQR: 27.0–31.0] *vs* 28.0 [25.0–30]; *p* < 0.001). WHOQOL-8 scores differed significantly between headache types (*p* < 0.001), being lowest among people with pMOH closely followed by those with migraine (Table 2). However, WHOQOL scores were negatively associated with frequency and intensity of headache (both *p* < 0.001); accordingly, among participants with headache, QoL was best in those with TTH (Table 2).

**Table 2:** Quality of life (WHOQOL-8 score) and willingness to pay according to headache type, frequency and intensity

	WHOQOL-8 score			Willingness to pay (NPR/month)		
	Mean ± SD	Median [IQR]	*p* ^a^	Mean ± SD	Median [IQR]	*p* ^a^
Headache type						
Migraine	26.7 ± 3.9	27.0 [24.0–29.0]	<0.001	1144 ± 2755	250 [100–250]	0.013
Tension-type headache	28.2 ± 3.7	28.0 [26.0–30.0]		1074 ± 2673	200 [60–200]	
Probable medication-overuse headache	25.7 ± 4.2	26.0 [23.0–29.0]		2031 ± 3960	500 [150–2000]	
Headache frequency (days/month)						
1–3	27.7 ± 3.9	28.0 [25.0–30.0]	<0.001	1056 ± 2760	200 [80–800]	<0.001
4–14	26.8 ± 3.7	27.0 (24.0–27.0)		1301 ± 2273	300 [150–1500)	
≥ 15	25.8 ± 4.0	26.0 (23.0–26.0)		1493 ± 3428	300 [150–1050)	
Headache intensity						
Not bad	28.7 ± 4.0	29.0 [26.0–32.0]	<0.001	1252 ± 4084	200 [50–900]	<0.001
Quite bad	27.6 ± 3.5	28.0 [26.0–30.0]		977 ± 2013	250 [100–800]	
Very bad	26.1 ± 4.1	26.0 [23.0–29.0]		1.322 ± 2690	300 [110–1200]	

*IQR* interquartile range, *NPR* Nepalese rupee; ^a^Kruskal-Wallis test

Participants with headache were willing to pay on average NPR 1134 ± 2760 (median 250 [IQR: 100–1000]) per month for effective headache care. WTP differed significantly (*p* = 0.013) between headache types, being highest among those with pMOH. WTP was positively associated with both frequency and intensity of headache (*p* < 0.001), and therefore least in TTH (Table 2).

### Disability

Among the 728 participants with migraine, 289 reported attacks of ≥24 h’ duration, with a mean of 57.1 h (D1) and a headache frequency of 2.38 days/month (HF1) (Table 3). Using the formula AF = HF/D (see Methods), we calculated AF for this group as AF1 = 2.38/(57.1/24), which we took to be 1. Among the 46 with pMOH, 17 reported headache that “never goes away”. We took their attack frequency (AF1) as 30, and duration (D1) as 24. All other calculations of disability are explained in the Methods section and set out in Table 3.

**Table 3:** Population-level disability (adults aged 18–65 years), by headache type

	Migraine	Tension-type headache	Probable medication-overuse headache
	*n* = 728 ≥ 24 h’ duration: *n* = 289; <24 h’ duration: *n* = 439	*n* = 863	*n*= 46 “never goes away”: *n* = 17; other: *n* = 29
Reported mean headache frequency [HF] (d/m)	2.3 (HF1 = 2.38, HF2 = 2.27)	1.8	23.1 (HF1 = 30, HF2 = 19.1)
Reported mean attack duration [D] (h)	33.6 (D1 = 57.1, D2 = 12.2)	16.0	12.1 (D1 = 24.0, D2 = 5.04)
Mean attack frequency [AF] (per month) (see text for explanation)	AF1 = 1, AF2 = 2.27	1.8	AF1 = 30, AF2 = 19.1
Mean absolute time in ictal state [T_abs_ = AF*D] (h/m)	Weighted mean of AF1*D1 and AF2*D2 = 39.36	AF*D = 28.8	Weighted mean of AF1*D1 and AF2*D2 = 326.8
Mean time in ictal state as proportion of total time T_pro_ = (T_abs_*12)/(365*24)] (%)	5.4	3.9	44.7
Disability weight [DW] (from GBD 2013 [3]	0.441	0.037	0.217
Mean disability per person with headache DIS_per_ = T_pro_*DW] (%)	2.4	0.15	9.7
Prevalence in adults aged 18–65 years [P] (%)	34.1	41.5	2.1
Disability in population [DIS_pop_ = DIS_per_ * P] (%)	0.81	0.06	0.20

For migraine, HF1, D1 and AF1 refer to those with attacks of ≥24 h’ duration, HF2, D2 and AF2 to those with attacks of <24 h’ duration; for probable medication-overuse headache, HF1, D1 and AF1 refer to those who report headache that never goes away, HF2, D2 and AF2 to those with headache of determinable duration; *D* days, *m* month, *h* hours

Mean DIS_per_ for migraine was 2.4 %; DIS_pop_ was 0.81 %. The corresponding values for TTH were substantially lower (0.15 and 0.06 %). For pMOH, however, DIS_per_ was very much higher, at 9.7 %, while DIS_pop_ was only 0.20 % because of the low prevalence.

### Headache-attributed lost time

Headache-attributed lost time in the preceding 3 months is presented by headache type in Table 4. All summed scores (total lost productive time, lost paid worktime and lost household worktime) had much higher means for pMOH than for migraine, which itself had means more than double those of TTH. However, it should be noted that, for all five individual items and all headache types, including pMOH, most medians were zero; this meant not only that distributions were very highly skewed, but also that at least half of respondents in most groups lost no time at all.

**Table 4:** Headache-attributed lost time in preceding 3 months for all headache and each headache type, by gender

	Total lost productive time		Lost paid worktime		Lost household worktime		Missed social and leisure activities	
	Mean ± SD	Median [IQR]	Mean ± SD	Median [IQR]	Mean ± SD	Median [IQR]	Mean ± SD	Median [IQR]
All headache								
all (*n* = 1794)	4.4 ± 9.2	1 [0–5]	1.7 ± 5.1	0 [0–0]	2.7 ± 5.9	0 [0–3]	0.5 ± 2.8	0 [0–0]
male (*n* = 689)	4.5 ± 9.6	0 [0–5]	2.5 ± 5.6	0 [0–3]	2.0 ± 5.9	0 [0–2]	0.7 ± 4.1	0 [0–0]
female (*n* =1105)	4.4 ± 9.0	1 [0–5]	1.2 ± 4.8	0 [0–0]	3.2 ± 5.9	0 [0–4]	0.4 ± 1.4	0 [0–0]
p^a^	0.91	0.034	<0.001	<0.001	<0.001	<0.001	0.046	0.10
Migraine								
all (*n* = 728)	5.4 ± 8.9	3 [0–7]	1.9 ± 4.6	0 [0–1]	3.5 ± 6.5	1 [0–5]	0.8 ± 4.1	0 [0–0]
male (*n* = 249)	7.1 ± 12.0	3 [0–9]	3.7 ± 6.3	0 [0–5]	3.4 ± 8.8	0 [0–3]	1.3 ± 6.5	0 [0–1]
female (*n* = 479)	4.5 ± 6.6	2 [0–6]	0.9 ± 3.0	0 [0–0]	3.5 ± 5.2	1 [0–5]	0.5 ± 1.7	0 [0–0]
p^a^	0.001	0.023	<0.001	<0.001	0.89	0.002	0.039	0.002
Tension-type headache								
all (*n* = 863)	2.2 ± 4.7	0 [0–3]	0.9 ± 3.1	0 [0–0]	1.3 ± 2.8	0 [0–1]	0.2 ± 0.9	0 [0–0]
male (*n* = 384)	2.0 ± 5.0	0 [0–2]	1.2 ± 3.6	0 [0–1]	0.8 ± 2.1	0 [0–0]	0.2 ± 0.8	0 [0–0]
female (*n* = 479)	2.3 ± 4.3	0 [0–2]	0.7 ± 2.5	0 [0–0]	1.6 ± 3.2	0 [0–2]	0.2 ± 0.9	0 [0–0]
p^a^	0.31	0.009	0.010	<0.001	<0.001	<0.001	0.76	0.94
Probable medication-overuse headache								
all (*n* = 46)	16.9 ± 25.9	6.5 [0–25.5]	8.0 ± 16.4	0 [0–6.2]	8.9 ± 13.6	0.5 [0–15]	1.2 ± 2.9	0 [0–7.3]
male (*n* = 11)	13.1 ± 14.7	7 [0–24]	10.2 ± 15.6	3 [0–24]	2.9 ± 6.2	0 [0–10]	2.8 ± 5.0	0 [0–2]
female (*n* = 35)	18.1 ± 28.1	6 [0–27]	7.3 ± 16.9	0 [0–5]	10.9 ± 14.7	5 [0–17]	0.7 ± 1.6	0 [0–0]
p^a^	0.44	0.87	0.60	0.23	0.015	0.089	0.18	0.17

*SD* standard deviation, *IQR* interquartile range; ^a^p was estimated using Student’s *t*-test for differences between means and Mann–Whitney *U*-test for medians

More household worktime (2.7 ± 5.9 days) was lost than paid worktime (1.7 ± 5.1 days), this difference being largely but not entirely attributable to migraine (Table 4). Regardless of headache type, males lost more paid worktime than females and the reverse was the case with household worktime.

From policy and economic perspectives, attention might focus on total lost productive time (4.4 days in 3 months) from all headache, since this was the mean loss for 85.4 % of the population. Assuming there were 13*5 working days in 3 months, this was a loss of 6.8 % for those affected, or 5.8 % for the population generally. However, 5.4 days lost in 3 months represented an 8.3 % individual loss for those with migraine. As they were 34.1 % of the population [14], this was a 2.8 % loss for the population generally.

## Discussion

This is the first nationwide population-based survey to estimate the burden of headaches disorders in Nepal, or in any country within the South-East Asia Region. The survey covered the whole country, recruiting a large representative sample through careful random selection and minimizing participation bias by achieving a very high participation rate (>99 %).

As is the case elsewhere in the world, we found burden levels were high in Nepal, on both individual and population levels. The symptom burden was itself large, carried by females more than males. On all measures, migraine imposed the greatest burden at population level while pMOH did so on affected individuals. This was reflected in impact on QoL. Participants with migraine spent, on average, 5.4 % of their time (equivalent to 20 days/year) in the ictal state, with headache of mean intensity 2.3, which would certainly be disabling. However, this 5.4 % is less than the 8.3 % lost productive time attributed to migraine, which says something about the relationship between these. Lost paid worktime was higher among males than females, but the reverse was true of lost household worktime.

Headache-attributed lost productive time is a well-validated measure of headache burden [17, 23, 25], yet it is not clear exactly what its determinants may be. It reflects not so much disability as behavioural response to impairment since, except in extreme cases, there is a degree of choice in either continuing or abandoning work when affected by headache [17]. In India, time spent by those with migraine in the ictal state (4.2 %) was also lower than the proportion of lost productive time (5.8 %) [26]. Rao et al. observed that it is in the nature of migraine that motivation and energy are lost, and that these symptoms, likely also to contribute to lost productivity, may for some time outlast what is described as the ictal state [26]. Additionally, our method of calculating time in ictal state for migraine was perhaps conservative in cases when attack duration was >24 h. In calculating lost productive time our assumption might also be questioned that there were only 65 working days in 3 months, especially for household work, but on the other hand this made no allowance for “holiday” time. It has also to be said that the high symptom burden of migraine in Nepal was not reflected in lost productive time to the extent that might be anticipated: lost paid and household worktime were both lower than in China [27], Georgia [28] and Zambia [8], although higher than in India [26] and much the same as in Russia [29].

In Nepal there are particular considerations that may be relevant. The proportion of people in paid employment is very low [13, 30]. This is especially true for women, who are predominantly engaged in agriculture; few have skilled manual jobs, and they are much less likely than men to be employed in professional, technical or managerial fields [30]. The work of many women in Nepal requires carriage of heavy loads by tumpline, weight-bearing on head and neck – not easy with any type of headache. Further, our enquiry might not have been clear that time spent, for example, in producing goods for home consumption, such as growing vegetables, should have counted in the context of the Nepalese economy as paid (“income”-generating) rather than household work. Therefore it is likely that lost productive worktime was underestimated, especially among women. The finding of greater lost productivity among males than females should be interpreted with caution in view of this and since the symptom burden was actually higher among females.

At population level, migraine caused most disability (0.81 %). We might compare this with 0.46 % in neighbouring India, where the survey used very similar methods but was conducted only in the southern State of Karnataka [26]. The difference approximately reflects the lower prevalence of migraine in India (25.2 %) [9]. The 0.81 % implies a reduction by this amount of population functional capacity. Whereas above we noted a 1.5-fold discrepancy between time spent in the ictal state of migraine and lost productive time, here there is much greater disparity: at population level, lost productive time due to migraine was 2.8 %, or 3.5 times the disability. Very similar disparities were found in India (3.3 times) [26] and in Zambia (also 3.3 times, despite a higher disability of 0.98 %) [8]. The determinants of lost productive time due to migraine may be unclear and probably complex, but we see evidence here, nonetheless, of constancy in its relationship to disability, which survives the influences of different behaviours among peoples from different cultures.

Information on WTP can, in theory, be used to estimate reasonable pricings and make economic forecasts before introducing new health-care services [31, 32]. We doubt its reliability for this purpose when gathered in surveys of this type, but see it more as a measure of overall burden [17]. As judged from the median WTP (in view of the skewed distribution), participants with headache were willing to pay NPR 250 (USD 2.50) per month for effective headache care, the amount correlating with headache type (pMOH > migraine > TTH), frequency and intensity. The absolute monetary value of this might be lower than has been reported from other, even low-income countries [28] but, put into local context, it would be a day’s earnings for an average Nepalese citizen [33]. As a measure of burden, this suggests heavy burden. As a reflection of what people with headache might be willing to invest in headache services, it signals need but probably not sufficient willingness (or ability) to pay.

Like all cross-sectional surveys, this study had limitations. Most importantly it relied on retrospective enquiry over 3 months, and therefore participants’ recall, to estimate impact of headache on work absence and productivity. The effect of recall error is uncertain, but it is more likely to have introduced random degrees of over- and underestimation than systematic error [24]. We focused on the most bothersome headache type in participants identifying more than one. In fact this avoided double-counting: although it might be possible in such a survey to diagnose multiple headache types, correctly attributing burden between them is not a realistic proposition. Among those with both migraine and TTH, the former would usually be the more bothersome [34]; this meant that some part of the burden of TTH was instead attributed to migraine, but from a public-health perspective this would have little consequence.

### Implications for Nepal

Headache disorders are not only common in Nepal but also highly burdensome: symptom burden is heavy, there is much consequential disability and substantial lost productivity. The economic cost is certain to be high. The remedy lies in better health care for headache; structured headache-care services are urgently needed in the country. However, Nepal is among the least developed countries in the world, with uneven distribution of its limited resources [11]. Government allocation to the health sector is also scarce; gaps in specialized health care exist at all levels [35]. Furthermore, Nepal’s rural hilly/mountainous geography poses great challenges to community health services. Creating new headache-care services would certainly demand investment of additional financial and human resources in the health sector; on the other hand, refraining from doing so will leave undiminished the burden of these highly prevalent but cost-effectively treatable brain disorders [36]. Structured headache services based in primary care [37] would be an efficient, effective, affordable and equitable model for Nepal, likely to be cost-saving [4]. Such services, appropriately adapted, could be implemented within the existing health-service structure of Nepal. This potential solution requires further research in order to inform political decision-makers. Doing nothing is almost certainly a more costly option.

## Conclusions

Headache disorders, very common in Nepal, are also highly burdensome at both individual and population levels. They cause disability and reduced functional capacity, with a substantial penalty in lost production. The remedy lies in better health care for headache; structured headache-care services are urgently needed in the country, and likely to be cost-saving.

## Abbreviations

ANOVA: Analysis of variance
DW: Disability weight
GBD: Global burden of disease
HALT: Headache-attributed lost time
HARDSHIP: Headache-attributed restriction, disability, social handicap and impaired participation
ICHD: International classification of headache disorders
IQR: Interquartile range
LTB: *Lifting the burden*
MOH: Medication-overuse headache
NPR: Nepalese rupee
pMOH: probable MOH
QoL: Quality of life
SD: Standard deviation
SEAR: South-East Asia Region
SPSS: Statistical package for social sciences
TTH: Tension-type headache
USD: United States dollar
WHOQOL-8: World Health Organization quality of life-8
WTP: Willingness to pay
YLD: Year of life lost to disability
